# Coordination of opposing sex-specific and core muscle groups regulates male tail posture during *Caenorhabditis elegans *male mating behavior

**DOI:** 10.1186/1741-7007-7-33

**Published:** 2009-06-22

**Authors:** Allyson J Whittaker, Paul W Sternberg

**Affiliations:** 1 Division of Biology/Howard Hughes Medical Institute, California Institute of Technology, Pasadena, CA 91125, USA

## Abstract

**Background:**

To survive and reproduce, animals must be able to modify their motor behavior in response to changes in the environment. We studied a complex behavior of *Caenorhabditis elegans*, male mating behavior, which provided a model for understanding motor behaviors at the genetic, molecular as well as circuit level. *C. elegans *male mating behavior consists of a series of six sub-steps: response to contact, backing, turning, vulva location, spicule insertion, and sperm transfer. The male tail contains most of the sensory structures required for mating, in addition to the copulatory structures, and thus to carry out the steps of mating behavior, the male must keep his tail in contact with the hermaphrodite. However, because the hermaphrodite does not play an active role in mating and continues moving, the male must modify his tail posture to maintain contact. We provide a better understanding of the molecular and neuro-muscular pathways that regulate male tail posture during mating.

**Results:**

Genetic and laser ablation analysis, in conjunction with behavioral assays were used to determine neurotransmitters, receptors, neurons and muscles required for the regulation of male tail posture. We showed that proper male tail posture is maintained by the coordinated activity of opposing muscle groups that curl the tail ventrally and dorsally. Specifically, acetylcholine regulates both ventral and dorsal curling of the male tail, partially through anthelmintic levamisole-sensitive, nicotinic receptor subunits. Male-specific muscles are required for acetylcholine-driven ventral curling of the male tail but dorsal curling requires the dorsal body wall muscles shared by males and hermaphrodites. Gamma-aminobutyric acid activity is required for both dorsal and ventral acetylcholine-induced curling of the male tail and an inhibitory gamma-aminobutyric acid receptor, UNC-49, prevents over-curling of the male tail during mating, suggesting that cross-inhibition of muscle groups helps maintain proper tail posture.

**Conclusion:**

Our results demonstrated that coordination of opposing sex-specific and core muscle groups, through the activity of multiple neurotransmitters, is required for regulation of male tail posture during mating. We have provided a simple model for regulation of male tail posture that provides a foundation for studies of how genes, molecular pathways, and neural circuits contribute to sensory regulation of this motor behavior.

## Background

**Figure 1:**
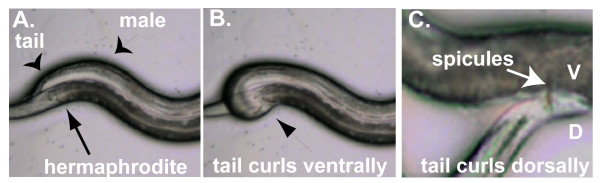
**Changes in male tail posture during steps of mating**. **(a) **Backing. Long black arrow, hermaphrodite, short black arrows, male and male tail. **(b) **Turning. Male tail curls ventrally, arrow. **(c) **Sperm transfer. V, D, ventral and dorsal sides of the male tail, respectively, arrow, male copulatory spicules.

Animals have developed a wide variety of motor behaviors to adapt to their changing environments. Impressive progress has been made in understanding how neurons and neural circuits act to regulate motor behavior [1]. However, less is known about how genes and molecular pathways contribute to neural circuits to regulate motor behaviors [2]. To better understand this, we studied male mating in *Caenorhabditis elegans*. Arguably the most complex motor behavior displayed by this organism, male mating can be separated into at least six sub-steps: response, backing, turning, vulva location, spicule insertion, and sperm transfer [3]. As *C. elegans *hermaphrodites do not actively cooperate in mating and continue crawling, to maintain contact with the hermaphrodite and successfully mate, the male undergoes a series of changes in body posture. The most dramatic changes occur in the male tail, which as it contains almost all of the sensory structures required for mating, must remain in contact with the hermaphrodite. A male begins mating by pressing the ventral side of his tail against the hermaphrodite while he backs along her side and searches for her vulva (Figure 1a). If the male reaches the end of the hermaphrodite without finding the vulva, he curls his tail sharply ventral, turns around the hermaphrodite, and searches on her other side (Figure 1b). After reaching the vulva and inserting his spicules, the male sometimes bends his tail dorsally (Figure 1c).

In addition to the neurons and muscles that the males share with hermaphrodites, ('core' neurons and muscles), each *C. elegans *male has an additional 89 sex-specific neurons and 41 sex-specific muscles, almost all of which are in its tail. Many of the sex-specific sensory neurons in mating behavior have known functions [3,4]. While less is known about how male-specific and core neurons and muscles interact to regulate mating behavior, sexual dimorphism of the core nervous system does contribute to mating behavior [5-7].

Serotonin release from the male-specific CP motor neurons has been proposed to activate the SER-1 serotonin receptor in male-specific muscles, causing ventral curling of the male tail [8-10]. However, other factors are also likely to regulate ventral curling. Males lacking male-specific muscles are still able to back along, and turn around a hermaphrodite, albeit clumsily, suggesting that core muscles partially control tail posture [8]. Likewise, males homozygous for mutations disrupting serotonin synthesis can still sometimes perform wild-type turns implying that neurotransmitters other than serotonin also act in tail curling. Finally, males lacking the CP neurons are able to turn sometimes while mating so other neurons must contribute to regulate tail posture. Meanwhile, dorsal curling of the male tail is not well understood.

Our studies showed that a balance of contraction of dorsal and ventral muscle groups maintains proper male tail posture. We showed that acetylcholine regulates both dorsal and ventral bending of the male tail, through both sex-specific and core muscle groups. Acetylcholine acts, in part, independently of serotonin to regulate ventral tail curling. Gamma-aminobutyric acid (GABA) is required for both dorsal and ventral curling of the male tail, suggesting that cross inhibition of muscle groups is important for proper regulation of tail posture. These studies provide insight into regulation of simple motor circuits and the basis of sexually dimorphic behaviors.

## Results

### Increased levels of synaptic acetylcholine induce male-specific changes in tail posture

**Figure 2:**
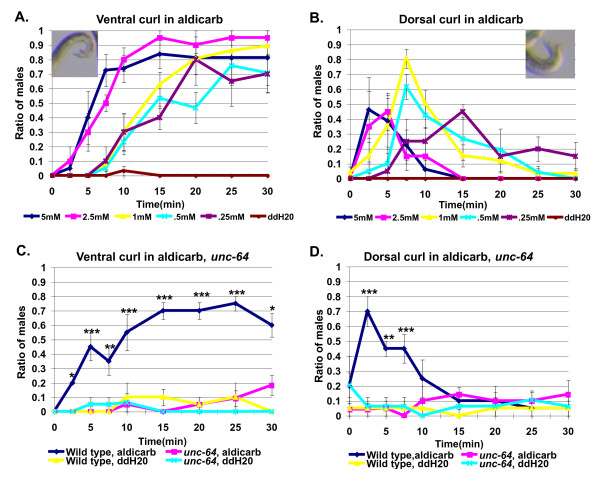
**The acetylcholinesterase inhibitor, aldicarb, induces ventral and dorsal curling of the male tail**. **(a) **Ventral and **(b) **dorsal tail curling of wild-type males in different concentrations of aldicarb (5 mM, *N *= 19; 2.5 mM, *N *= 20; 1.0 mM, *N *= 26; 0.5 mM, *N *= 21; 0.25 mM, *N *= 20; ddH20, *N *= 30). Insert figure shows the extent of tail curling (anterior is left, posterior is right, image has been flipped in (a)). **(c) **Comparison of ventral and **(d) **dorsal tail curling in wild-type control vs. *unc-64 (e246) *mutant males in 1 mM aldicarb and ddH20 (wild-type 1 mM aldicarb, *N *= 20; *unc-64 (e246) *1 mM aldicarb, *N *= 21; wild-type ddH20, *N *= 20; *unc-64 *ddH20, *N *= 19). *P < 0.05, **P < 0.005, ***P ≤ 0.0005. Error bars indicate +/- standard error of the mean.

Acetylcholine acts as an excitatory neurotransmitter at the *C. elegans *neuromuscular junction and regulates locomotion, pharyngeal pumping, spicule insertion, and egg-laying behavior [11-16]. To examine the role of acetylcholine in the regulation of male tail posture, we bathed males in aldicarb, an acetylcholine esterase inhibitor that increases endogenous acetylcholine at synapses [17]. Aldicarb makes *C. elegans *males curl their tails either ventrally or dorsally, with tail curling occurring earlier at higher concentrations than lower concentrations. Dorsal curling of the male tail is seen before ventral curling and decreases dramatically once ventral tail curling begins (Figure 2a and 2b). Tail curling was not seen in water alone (Figure 2a and 2b). If tail curling in response to aldicarb results from more acetylcholine at synapses, lowering synaptic vesicle release should block aldicarb-induced tail curling. To test this, we examined males homozygous for a mutation in *unc-64*. *unc-64 *encodes *C. elegans *syntaxin, a component of the synaptic vesicle fusion machinery [18,19]. In 1 mM aldicarb, a concentration that normally strongly induces dorsal and ventral tail curling, *unc-64(e246) *mutant males showed a dramatic decrease in both ventral and dorsal curling (Figure 2c and 2d). Thus, aldicarb-induced tail curling results from increased levels of endogenous acetylcholine.

**Figure 3:**
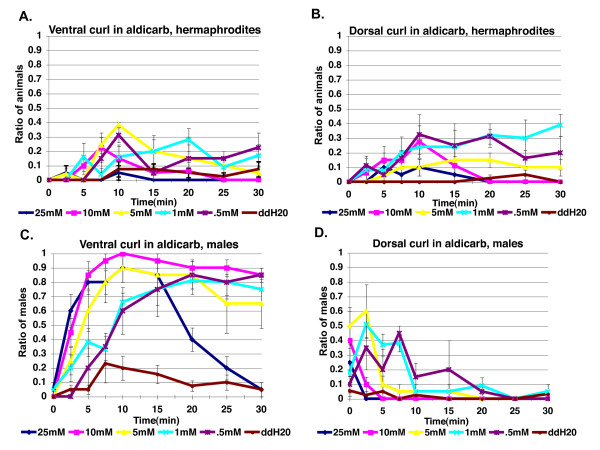
**The acetylcholinesterase inhibitor, aldicarb, does not induce significant tail curling in hermaphrodites**. **(a) **Ventral and **(b) **dorsal curling in hermaphrodites (25 mM, *N *= 20 for time points 0 to 15; *N *= 19 for time points 20 to 30; 10 mM, *N *= 19 for time points 0 to 15; *N *= 18 for time points 20 to 30; 5 mM, *N *= 21; 1 mM, *N *= 25 for time points 0 to 20; *N *= 23 for time points 25 to 30; 0.5 mM aldicarb, *N *= 19; ddH20, *N *= 40). **(c) **Ventral and **(d) **dorsal tail curling in males assayed on the same day as hermaphrodites (25 mM, *N *= 20; 10 mM, *N *= 20; 5 mM, *N *= 20; 1 mM, *N *= 21; 0.5 mM, *N *= 20; ddH20, *N *= 38). Error bars indicate +/- standard error of the mean. Males show a significantly higher amount of ventral tail curling at several time points. Males show a significantly higher amount of dorsal tail curling versus hermaphrodites in aldicarb at early time points: 25 mM/0 min, 1 mM/5 min, *P *< 0.05; 10 mM/0 min, *P *< 0.005; 5 mM/0 min,2.5 min, 1 mM/2.5 min. *P *< 0.0005).

To test if cholinergic regulation of tail postures is specific to males, we compared the response of males and hermaphrodites to five different aldicarb concentrations ranging from 25 mM to 0.5 mM. Although occasional slight ventral curling was seen at the most posterior end of the hermaphrodite, it did not occur with either the same degree of bending or the high frequencies seen in males at any concentration. At early time points, dorsal tail curling was seen at significantly higher levels in males than in hermaphrodites, demonstrating that there is sexually dimorphic regulation of dorsal curling (Figure 3). Therefore, cholinergic pathways regulate male-specific changes in tail posture.

### Acetylcholine regulates male tail posture via both core and sex-specific muscles

**Figure 4:**
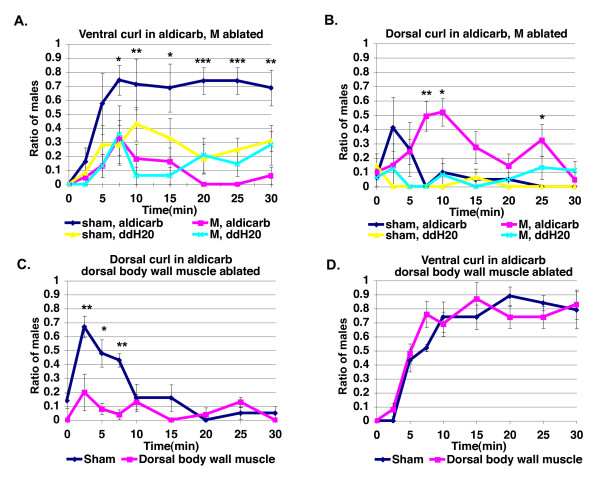
**Male-specific muscles are required for ventral curling, and dorsal body wall muscles are required for dorsal tail curling in response to aldicarb**. **(a) **and **(b) **Percentage of males with tail curls in 1 mM aldicarb is compared for M cell- and sham-ablated males (sham-ablated 1 mM aldicarb, *N *= 17; M cell-ablated, 1 mM aldicarb, *N *= 17; sham-ablated ddH20, *N *= 16; M cell-ablated ddH20 *N *= 17). **(a) **Ventral. **(b) **Dorsal. **(c) **and **(d) **Percentage of males with tail curls in 1 mM aldicarb is compared for body wall muscle and sham-ablated males. Dorsal body wall-ablated, *N *= 25 for time points 0 to 7.5, and *N *= 23 for time points 10 to 30. Sham-ablated males, *N *= 21 for time points 0 to 7.5, and *N *= 19 for time points 10 to 30. **(c) **Dorsal. **(d) **Ventral. *P < 0.05, **P < 0.005, ***P < 0.0005 Fisher's exact test. Error bars indicate +/- standard error of the mean.

*C. elegans *males have 41 sex-specific muscles generated from one postembryonic precursor cell, the M cell [9]. In the absence of these muscles, males are still able to respond, back, turn, and find the vulva, but they do so with decreased coordination and efficiency, suggesting that in addition to sex-specific muscles, sexually dimorphic control of the core muscles is important for regulation of male tail posture [8]. To determine if cholinergic regulation of male tail posture requires male-specific muscles, we ablated the M precursor cell using laser microsurgery and examined the male's ability to respond to 1 mM aldicarb. M cell-ablated males were no longer able to make a ventral curl in response to aldicarb (Figure 4a). In a small number of animals, a slight ventral bend and occasionally a full curl was seen, suggesting that core muscles, perhaps the ventral body wall muscles, also contribute to ventral curling in response to acetylcholine, but do not play as significant a role as M cell-derived muscles.

Dorsal curling in response to aldicarb, in contrast, was raised rather than lowered at later time points by the absence of male-specific muscles (Figure 4b). We therefore ablated dorsal body wall muscles in the tail region of the male and found that dorsal tail curling is eliminated in most males indicating that acetylcholine acts through these core muscles (Figure 4c). The low level of tail curling seen in some animals may be due to incomplete dorsal body wall muscle ablation. Ventral curling, in contrast, is not disrupted (Figure 4d). Thus, acetylcholine regulates male tail posture through both male-specific muscles and sexually dimorphic control of core body wall muscles.

To determine whether posterior dorsal body muscles were necessary for male tail posture control during mating, we ablated the muscles in males and examined their mating behavior. Ablated males over-curled their tails ventrally when mating (Table 1). When they first touch the hermaphrodite, ablated males often respond by curling around the hermaphrodite. When backing, ablated males either curl their tails slightly ventrally, resulting in them backing on the lateral sides of the hermaphrodite, or curl so much that they turn early before reaching the end of the hermaphrodite. Ablated males turned more poorly than sham-ablated males, as determined by the percentage of males having a greater number of good turns versus poor turns, primarily because ablated males did more early turns. However, ablated males were still able to insert their spicules into hermaphrodites, and keep their spicules inserted long enough to transfer sperm (data not shown).

**Table 1:** Backing and turning phenotypes of dorsal body wall-ablated males.

**Line**	**Curl around hermaphrodite**	**Back on lateral side**	**Turns early**	**Number of good turns > number of bad turns**
Dorsal body wall-ablated	22.2% *N *= 27	14.8% *N *= 27	37.5% *N *= 24	54% *N *= 24

Sham-ablated	4.3% *N *= 23 *P *= 0.1068	0% *N *= 23 *P *= 0.1147	5% *N *= 20 *P *= 0.013	75% *N *= 20 *P *= 0.2125

### Levamisole receptors are required for proper tail posture control

**Figure 5:**
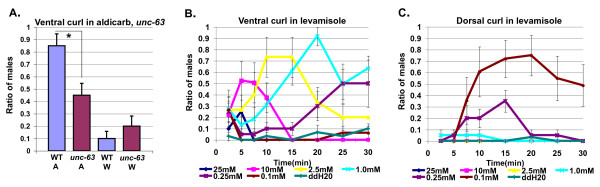
**Acetylcholine acts in part through levamisole receptors to regulate tail posture**. **(a) **Ventral curling in wild-type versus *unc-63(e384) *males in 1 mM aldicarb. The highest amount of tail curling at the 25- and 30-minute time points (the time points where the most ventral tail curling was seen) for each trial was averaged. For all genotypes and conditions *N *= 20 *P < 0.05 Fisher's exact test. **(b) **Ventral and **(c) **dorsal male tail curling in levamisole (25 mM, *N *= 20; 10 mM, *N *= 21; 2.5 mM, *N *= 15; 1.0 mM, *N *= 21; 0.25 mM, *N *= 20; 0.1 mM, *N *= 19; ddH20, *N *= 30). Error bars indicate +/- standard error of the mean. W = water; A = aldicarb.

*C. elegans *has at least 29 nicotinic and 3 muscarinic acetylcholine receptor subunits as well as acetylcholine-sensitive chloride channels [20-22]. To dissect the cholinergic pathways mediating male tail posture, we examined the effects of mutations in nicotinic acetylcholine receptor subunits on tail curling in response to aldicarb. The nicotinic acetylcholine receptor α subunit, UNC-63, was required for efficient ventral tail curling in response to aldicarb (Figure 5a). Since *unc-63 *mutant males also show an increased level of dorsal tail curling in water alone, it was not possible to determine if *unc-63 *is also required for dorsal tail curling.

**Figure 6:**
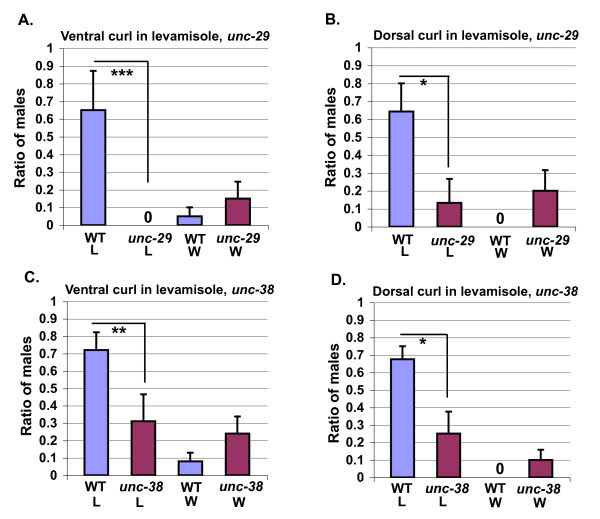
**The levamisole receptor subunits *unc-29 *and *unc-38 *are required for ventral and dorsal tail curling in response to levamisole**. The highest amount of tail curling at the 15- to 20-minute time points for each trial was averaged. **(a) **A comparison of ventral tail curling of wild-type males and *unc-29 (e1072) *mutant males in 1 mM levamisole (*N *= 20 for all genotypes and conditions). **(b) **A comparison of dorsal tail curling of wild-type versus *unc-29(e1072) *mutant males in 0.1 mM levamisole (wild-type 0.1 mM levamisole, *N *= 16 for all other conditions, *N *= 15). **(c) **A comparison of ventral tail curling in wild-type versus *unc-38(sy576) *mutant males in 1 mM levamisole (wild-type, 1 mM levamisole, wild-type ddH20, and *unc-38 *ddH20, *N *= 25; *unc-38*, 1 mM levamisole, *N *= 24). **(d) **A comparison of dorsal tail curling in wild-type versus *unc-38(sy576) *mutant males in .1 mM levamisole (wild-type 0.1 mM levamisole, N = 19 for all other genotypes and conditions, N = 20). W = water; L = levamisole.

UNC-63 is one of five subunits of the levamisole receptor which itself is one of two nicotinic receptors required for activation of *C. elegans *body wall muscles [23]. Two levamisole receptor α-subunits, UNC-38 and UNC-63, as well as a non-α subunit, UNC-29, are required for body wall muscle paralysis in hermaphrodites by levamisole, whereas mutations in the non-α LEV-1 and α LEV-8 subunits merely diminish paralysis by levamisole [24-27]. We thus bathed males in the nicotinic agonist levamisole to test levamisole receptor function in tail posture. Like aldicarb, levamisole induces ventral or dorsal curling of the male tail in a dose and concentration-dependent manner (Figure 5b and 5c). These results differed from those reported by Loer and Kenyon [8], who found that males bathed in levamisole only showed a slight dorsal tail curl. The difference in our results may be due to the fact that we diluted our drug in water rather than M9, in which levamisole is significantly less potent [28]. Neither dorsal nor ventral curling in response to levamisole are seen in hermaphrodites (data not shown). Mutations in *unc-29 *and *unc-38 *nearly eliminated dorsal curling in response to 0.1 mM levamisole, and ventral tail curling in response to 1 mM levamisole (Figure 6). These concentrations were chosen as they were the concentrations at which we saw the highest amount of ventral and dorsal curling, respectively. Differences in the sensitivity of dorsal and ventral tail curling may reflect differences in the pathways regulating these behaviors.

**Figure 7:**
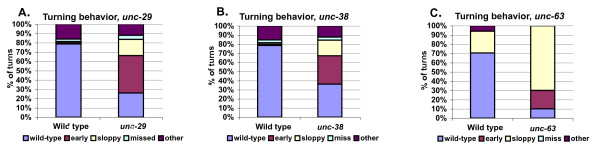
***unc-29*, *unc-38 *and *unc-63 *mutant males have poor turning behavior**. **(a) **Ninety-two turns of 34 males were averaged for *unc-29(e1072)*; 75 turns of 39 males were averaged for wild-type. **(b) **Fifty-eight turns from 17 males were averaged for *unc-38(sy576)*; 65 turns from 29 males were averaged for wild-type. **(c) **Ten turns from five males were averaged for *unc-63*(*e384*); 17 turns from six males were averaged for wild-type.

Since mutations of its receptors block levamisole's effect on tail posture, we examined response, backing, and turning behavior of *unc-29*, *unc-38*, and *unc-63 *mutant males to see if these genes are needed under biological conditions. Consistent with an inability to bend the tail ventrally, *unc-29*, *unc-38*, and *unc-63 *males have difficulty keeping the ventral side of their tail in contact with hermaphrodites during backing. Also, *unc-29*, *unc-38*, and *unc-63 *mutant males showed turning phenotypes that are similar to males in which the M cell is ablated (Figure 7). *unc-29*, *unc-38 *and *unc-63 *mutant males show a higher number of sloppy turns (male makes a wide arc with his tail at the end of the hermaphrodite before re-establishing contact with her opposite side), missed turns (male fails to turn at the end of the hermaphrodite), tip turns (male turns around the tip of the hermaphrodite), and stutter turns (male repeatedly initiates but does not complete a turn) [5,8]. We also saw phenotypes similar to those in which the dorsal body wall muscles are ablated. *unc-38*, *unc-29*, and *unc-63 *mutant males turned early more often than wild-type males, and were more likely to curl around the hermaphrodite when initially responding, and back on the lateral sides of the hermaphrodite (Figure 7 and data not shown).

**Figure 8:**
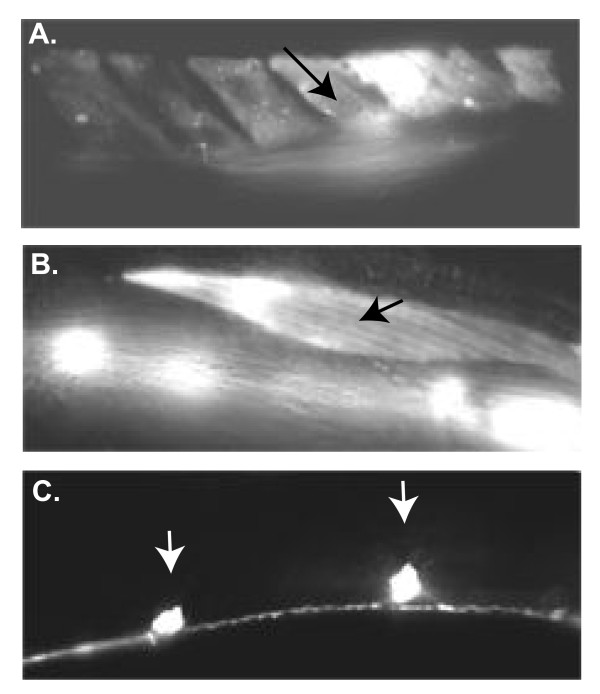
***unc-29 *is expressed in both muscles and neurons to regulate tail curling**. **(a)-(c) ***unc-29*::dsRed expression. **(a) **Diagonal muscles. Arrow, a diagonal muscle. **(b) **body wall muscles. Arrow, a body wall muscle. **(c) **Ventral cord motor neurons, arrows.

In hermaphrodites *unc-38*, *unc-29*, and *unc-63 *are expressed both in neurons and in body wall and hermaphrodite-specific muscles [14,25,26,29]. In males, *unc-38 *is expressed in the male-specific muscles and body wall muscles, but its neuronal expression is not well described [30]. We thus generated an *unc-29*::dsRed transcriptional fusion construct and found that *unc-29 *is expressed in male-specific muscles such as the diagonal muscles, as well as in body wall muscles (Figures 8a and 8b). Since the levamisole receptor is required in the body wall muscle for proper locomotion, it may act in dorsal body wall muscles in males to regulate dorsal tail curling. Expression in the diagonal muscles suggests that the levamisole receptor may act, in part, in diagonal muscles to regulate ventral tail curling. *unc-29*::dsRed is also seen in neurons in the ventral cord and head and thus may also act in neurons to regulate male tail posture (Figure 8c and data not shown).

### Acetylcholine can act independently of serotonin to regulate ventral tail curling

**Figure 9:**
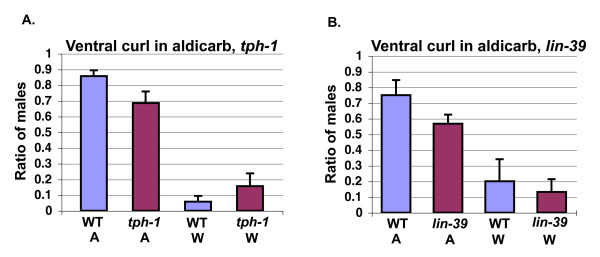
**Acetylcholine can act independently of serotonin to induce ventral and dorsal tail curling**. **(a) **Comparison of tail curling in aldicarb of wild-type versus *tph-1 (mg280) *males (wild-type and *tph-1*, aldicarb, *N *= 35; wild-type ddH20, *N *= 34; *tph-1 *ddH20, *N *= 32). **(b) **Comparison of tail curling in response to aldicarb in *lin-39 *(*n1760)*, versus wild-type males (wild-type, aldicarb, *N *= 20; *lin-39*, aldicarb, *N *= 18; *him-5*, ddH20, *N *= 20; *lin-39*, ddH20, *N *= 18). For (a) and (b), the highest amount of tail curling at the 20- to 25-minute time points was averaged. W = water, A = aldicarb, WT = wild-type.

Since males bathed in serotonin curl their tails ventrally [8], we wanted to examine if serotonin is required for cholinergic regulation of male tail curling. *tph-1 *mutant males, deficient in tryptophan hydroxylase and hence serotonin, showed a small but not significant decrease in ventral tail curling in aldicarb (Figure 9a) [31]. Dorsal tail curling was also largely unaffected in *tph-1 *mutant males (data not shown).

To test serotonergic CP motor neurons for function in acetylcholine-induced ventral tail curling, we examined the ability of *lin-39 *mutant males to respond to aldicarb. In *lin-39 *mutant males, CP neurons 1–4 die or are necrotic, while CP neurons 5 and 6 take on a more posterior fate, and *lin-39 *mutant males have a turning defect similar to that of males in which the CP neurons have been ablated [8,32-34]. We found that ventral curling of *lin-39 (n1760) *mutant males in response to aldicarb was lower, but not significantly so (Figure 9b). Thus, acetylcholine largely acts downstream or in parallel to serotonin and cholinergic neurons act in parallel to the CP motor neurons to regulate ventral tail curling.

### GABA is required for both dorsal and ventral curling of the male tail

**Figure 10:**
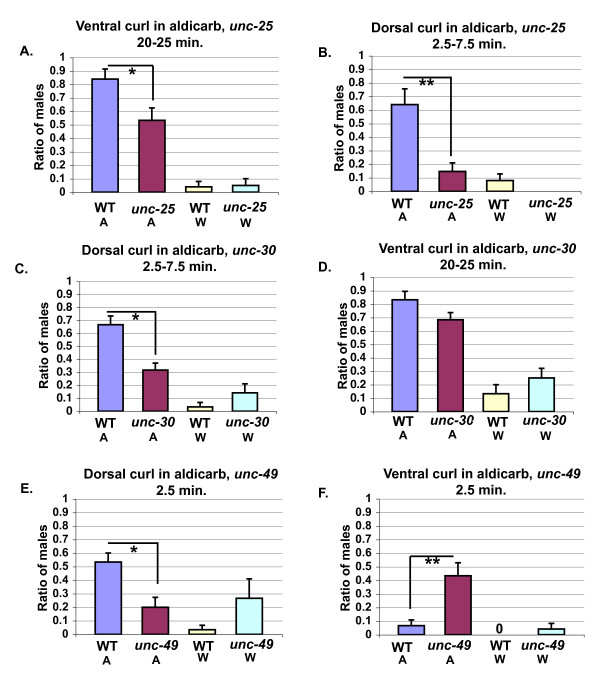
**Acetylcholine acts in part through GABAergic neurons to regulate male tail posture**. **(a) **and **(b) ***unc-25 *versus wild-type males in aldicarb: wild-type, aldicarb, *N *= 25; *unc-25*, aldicarb, *N *= 21; wild-type, ddH20, *N *= 25; *unc-25*, ddH20 *N *= 20. **(a) **Ventral. The highest amount of tail curling at the 20- to 25-minute time points for each trial was averaged. **(b) **Dorsal. The highest amount of tail curling at the 2.5- to 7.5-minute time points for each trial was averaged. **(c) **and **(d) ***unc-30 *versus wild-type in aldicarb. wild-type aldicarb and ddH20 *N *= 30; *unc-30 *aldicarb and ddH20 *N *= 29. **(c) **Dorsal. The highest amount at the 2.5- to 7.5-miute time points for each trial was averaged. **(d) **Ventral. The highest amount at 20- to 25-minute time points for each trial was averaged. **(e) **and **(f) **Mutations in the *unc-49 *gamma-aminobutyric acid receptor disrupt tail curling at only the 2.5-minute time point. wild-type and *unc-49*, aldicarb *N *= 30;wild-type, ddH20, *N *= 30; *unc-49*, ddH20, *N *= 29. **(e) **Dorsal tail curling at the 2.5-minute time point. **(f) **Ventral tail curling at the 2.5-minute time point. *P < 0.05, **P < 0.005. Fisher's exact test. Error bars indicate +/- standard error of the mean. W = water, A = aldicarb, WT = wild-type.

During hermaphrodite locomotion, GABA is released from D-type motor neurons and alternately inhibits dorsal and ventral body wall muscles via the inhibitory GABA receptor (GABAR), UNC-49 [35,36]. To test if GABA is also required for regulation of male tail posture, we first examined the response of *unc-25 *mutant males, deficient in glutamic acid decarboxylase, to aldicarb [37]. Mutations in *unc-25 *significantly decreased both dorsal and ventral aldicarb-induced curling (Figure 10a and 10b). Thus, acetylcholine acts in part through GABAergic neurons to regulate both ventral and dorsal tail curling. In this assay, dorsal curling is disrupted to a greater extent than ventral tail curling by mutations in *unc-25*. This could suggest that GABA plays a more critical role in cholinergic regulation of dorsal tail curling, or that, in this assay, fewer muscle groups act to bend the tail dorsally, and thus inhibition of ventral muscle groups is more critical.

To test if D-type motor neurons are required for tail curling, we examined aldicarb-induced tail curling of males homozygous mutant for *unc-30*, a homeodomain containing transcription factor that is required for the terminal differentiation of the D-type motor neurons [38]. *unc-30 *mutant males showed a significant decrease in dorsal tail curling, indicating that acetylcholine may act in part through the D-type neurons to promote dorsal tail curling (Figure 10c). Ventral tail curling, in contrast, is largely unaffected in *unc-30 *mutant males suggesting that other GABAergic neurons are involved (Figure 10d).

*unc-49 *mutant males show a small but significant decrease in aldicarb-induced dorsal tail curling in response to aldicarb at only the 2.5 minute time point and a significant increase in ventral tail curling (Figure 10e and 10f). Thus, although acetylcholine may partly act through UNC-49 to regulate dorsal tail curling, other GABARs are involved. As we saw an increase in ventral tail curling at the 2.5 minute time point, it suggests that acetylcholine regulates dorsal tail curling, in part by inhibiting ventral muscle groups.

While mating, *unc-49 *mutant males had difficulty maintaining proper tail posture when first responding to, and backing along, a hermaphrodite due to ventral over-curling of the tail (Table 2). Males curled their tails ventrally in on themselves when initially responding to, and backing along, hermaphrodites and often turned early. *unc-49 *mutant males also over-curled their tails dorsally. When wild-type males have inserted their spicules into the vulva, they often curl their tails dorsally; *unc-49 *mutant males often curl dorsally to such a great extent that they flip over and lose contact with the hermaphrodite (Table 2). This suggests that inhibition of ventral and dorsal tail curling via the *unc-49 *receptor is required to maintain proper tail posture during mating.

**Table 2:** Mating phenotypes of *unc-49(e407) *mutant males.

**Line**	**Mutant response**	**Mutant backing**	**Turns early**	**Mutant vulva location**	**Improper body posture at vulva**
Wild-type	0% *N *= 7	0% *N *= 7	0% *N *= 7	0% *N *= 7	0% *N *= 7

*unc-49(e407)*	71.4% *N *= 9 *P *= 0.0337	57.1% *N *= 7 *P *= 0.0699	57.1% *N *= 7 *P *= 0.0699	0% *N *= 7	85.7% *N *= 7 *P *= 0.0047

## Discussion

**Figure 11:**
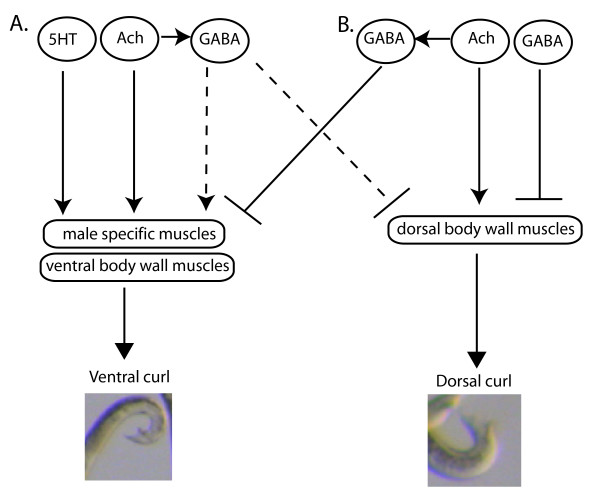
**Model for regulation of male tail posture**. Proper tail posture is maintained through a balance of ventral and dorsal curling of the male tail. **(a) **Ventral tail curling. Acetylcholine acts through male-specific and likely ventral body wall muscles to curl the tail ventrally. Acetylcholine acts together with serotonin, likely in part by acting directly on male-specific muscles to promote ventral tail curling. Acetylcholine also acts through GABAergic neurons to promote ventral tail curling by activating ventral muscles and/or by inhibiting dorsal muscles. **(b) **Dorsal tail curling. Acetylcholine acts through dorsal body wall muscles to promote dorsal tail curling. Acetylcholine also acts through GABAergic neurons to promote dorsal tail curling in part by inhibiting ventral tail curling via the *unc-49 *receptor. Dorsal tail curling, seen when males have their spicules inserted into the vulva, is inhibited by gamma-aminobutyric acid through the *unc-49 *receptor.

Control of *C. elegans *tail posture during mating provides an excellent model system for understanding adaptation of motor behavior in response to sensory input at both the molecular and neural circuit level. Our results have provided a simple model for control of male tail posture during mating and suggest proper regulation of male tail posture requires coordination of opposing muscle groups (Figure 11). Ventral curling of the male tail is seen when a male presses the ventral side of his tail into the hermaphrodite when responding to contact and backing, and greater ventral tail curling is seen when a male turns around a hermaphrodite. Male-specific muscles, together with core muscles, likely the ventral body wall muscles, are required to regulate ventral tail curling [8]. We have shown that the core dorsal body wall muscles are also important for regulating proper tail posture during mating and that the core dorsal body wall muscles regulate dorsal bending of the male tail in response to acetylcholine. During backing, the opposing dorsal body wall muscles may be relaxed, or alternatively, they may contract in order to counterbalance ventral bending of the male tail. Our results favor the later hypothesis. Males that lack the most posterior dorsal body wall muscles often over-curl their tails ventrally, resulting in them curling around the hermaphrodite when first responding, backing on the lateral sides of the hermaphrodite and turning early. In mammals, co-contraction of opposing muscle groups is used to stiffen a limb, seen for example, when an arm is stiffened right before catching a falling ball, and it is also used to prevent over-contraction of the opposing muscle groups. In some species of nematodes, males mate by curling around the hermaphrodite in a pattern similar to what is sometimes seen when *C. elegans *males with dorsal body wall muscles ablated first respond to hermaphrodites. Our results suggest that the increase in ventral bending seen in other species may result, in part, from less opposing activity of the dorsal body wall muscles.

Our results favor a model where serotonergic neurons act with cholinergic neurons to promote contraction of male-specific muscles and ventral tail curling. First, bathing males in the acetylcholine-esterase inhibitor aldicarb induces ventral curling of the male tail and this requires the male-specific muscles, which are necessary for efficient response and turning behavior [8]. In addition, mutations in levamisole receptor subunits disrupt response and turning behaviors, consistent with acetylcholine regulating male tail posture during mating. We cannot rule out the possibility that due to contextual and temporal factors, there are some differences between the aldicarb assay and tail posture regulation during mating. Previous studies suggest a model where serotonin, released from the CP motor neurons, acts on the SER-1 receptor in the male-specific diagonal muscles to promote ventral curling of the male tail [8,10]. We showed that a mutation that eliminates the CP 1–6 motor neurons does not significantly decrease aldicarb-induced ventral tail curling suggesting that there are cholinergic neurons that act, in part, in parallel to the CP motor neurons to regulate ventral tail curling. As levamisole receptor subunits are expressed in male-specific muscles, it is likely that these muscles are regulated by both acetylcholine and serotonin. It is likely that further study will reveal additional levels of complexity in cholinergic and serotonergic regulation of male tail posture. Consistent with this, our results showed that levamisole receptors are also expressed in neurons. Also, other acetylcholine receptor subunits have been shown to be expressed in neurons required for backing and/or turning behavior and there is evidence that muscarinic acetylcholine receptors regulate male tail posture [30,39]. In egg-laying behavior, acetylcholine and serotonin act in an excitatory manner to regulate contraction of vulva muscles. However, they also act in an inhibitory manner through motor neurons to inhibit egg-laying [40]. The sex-specific muscles and some neurons involved in egg-laying and ventral tail curling have the same precursor in males and hermaphrodites, providing an interesting model for studying development of sex-specific neural circuits on the neuronal and molecular level.

GABA activity is required to maintain proper tail posture during several steps of mating behavior. Males with mutations in the gene encoding the GABAR, UNC-49, bend their tails ventrally to too great an extent when responding to and backing along hermaphrodites. Also, males with mutations in *unc-49 *excessively bend their tails dorsally when spicules are inserted into the vulva, often to such an extent that males flip over and lose contact with the hermaphrodite. GABAergic regulation of male tail posture is in part regulated by acetylcholine. Males carrying a mutation that eliminates GABA synthesis, *unc-25*, show decreased dorsal and ventral tail curling in response to increased levels of acetylcholine. Mutations in *unc-49 *do not decrease dorsal tail curling to the same extent as mutations that eliminate GABA synthesis, and increase ventral tail curling at the 2.5-minute time point, suggesting other GABARs are additionally involved. There are other GABARs in the *C. elegans *genome, including an excitatory GABAR, *exp-1 *[41,42]. Males homozygous for a mutation in *unc-30*, required for core GABAergic D-type neuron specification, show a decrease in aldicarb-induced dorsal tail curling. In hermaphrodites the cholinergic ventral cord motor neurons synapse on to the D-type neurons to regulate cross inhibition of body wall muscles, and thus, they are good candidates for regulation of male tail posture. However, we cannot rule out the possibility that, in males, additional neurons are specified by *unc-30*, and these may be required. A mutation in *unc-30 *does not disrupt dorsal curling as much as *unc-25 *mutations and does not effect ventral curling suggesting other, possibly male-specific GABAergic neurons, are required for cholinergic regulation of tail curling.

Our results have demonstrated that *C. elegans *male tail posture is a complex motor behavior requiring coordination of different muscle groups and involving several neurotransmitters. The relative simplicity of the *C. elegans *nervous system makes it possible to understand regulation of this motor behavior on both a molecular and neural circuit level. There is increasing evidence that basic circuit principles are shared between *C. elegans *and higher organisms and thus our studies will provide insight into control of adaptive motor behaviors in higher organisms [43,44].

## Conclusion

Male mating behavior is a complex behavior requiring coordinated regulation of multiple muscle groups in response to sensory input, and provides a powerful model to understand how genes and molecular pathways contribute to neural circuits that regulate motor behavior. Our results have provided insight into how the *C. elegans *male regulates his tail posture during mating to enable him to maintain contact with the hermaphrodite and successfully mate. We showed that proper male tail posture is maintained through a balance of activity of opposing dorsal and ventral muscle groups, and we provided a relatively simple model for neurotransmitters, receptors, neurons and muscles required for this behavior. Our results demonstrated that acetylcholine regulates both ventral and dorsal curling of the male tail. For ventral curling, acetylcholine acts partly in parallel with serotonin to regulate activity of the male-specific muscles. Acetylcholine acts in part through the nicotinic levamisole receptors, likely acting in male-specific muscles but also neurons. Acetylcholine-mediated ventral tail curling requires GABA. However, acetylcholine-induced ventral tail curling does not require the body wall muscle GABAR, UNC-49, and thus other inhibitory or excitatory GABARs are involved in promoting ventral curling of the male tail. Dorsal curling, in contrast to ventral curling, requires the core dorsal body wall muscles. Absence of these muscles results in the male over-curling his tail ventrally while backing along the hermaphrodite, which suggests that contraction of dorsal muscles may be required to counterbalance ventral tail muscles. Acetylcholine-mediated dorsal tail curling requires GABA and likely the inhibitory D-type motor neurons. However, mutations in the GABAR, UNC-49, only partially decreased acetylcholine-induced dorsal tail curling, which suggests that other GABARs are involved. Together, these results present a more detailed understanding of regulation of male tail posture during mating, and provide a basis for future studies that will yield new insights into genetic and neuronal regulation of motor behaviors.

## Methods

### Strains

All strains were cultured using standard methods [45]. To increase the number of males we used a *him-5(e1490) *mutation in the background of the wild-type N2 strain in the following strains: *unc-29(e1072)*, *unc-38(sy576), tph-1(mg280)*, *lin-39(n1760)*, *unc-64(e246)*, and *unc-30(e191)*. To generate males in the following strains, hermaphrodites were heat shocked at 29°C for approximately 10 hours and then the resulting male and hermaphrodite progeny were crossed to generate males: *unc-63(e384)*, *unc-25(e156)*, and *unc-49(e407)*.

### Behavioral assays

As there is day-to-day variation in behavioral assays, in all assays, control animals were assayed on the same day as mutant or ablated males for comparison.

#### Mating assays

Mating behavior was observed using a Wild M5A microscope at ×25 and ×50 magnification. Males were isolated from hermaphrodites as L4s and used the following day. Males were only assayed one time and then destroyed. An exception to this was that M cell-ablated and sham-ablated control males were used 16 to 32 hours after being isolated as L4s. Also, these males were assayed two times with at least a 1-hour recovery period between assays. Males were assayed for their ability to mate with partially paralyzed *unc-31(e169) *hermaphrodites. Response behavior was recorded as follows: the total amount of time it took males to respond to a hermaphrodite was noted (initial response). Also, the length of time males spent responding to a hermaphrodite before leaving or finding the vulva was noted (continued response). Backing behavior was assayed as follows: males were observed for their ability to back along the hermaphrodite. In addition to noting the ability to back along the hermaphrodite, it was also noted if males instead of backing along the dorsal and ventral sides of the hermaphrodite, backed along the lateral sides of the hermaphrodite. Turning behavior was assayed as follows: the number of bad turns was noted. Turns were categorized as missed, sloppy, tip, or stutter as described in Liu *et al*. [5] and Loer and Kenyon [8]. In addition, we saw turns that were initiated before the normal turning point of the male, which we labeled 'early'. If a male executed a turn and then pulled back and executed another turn instead of continuing to back on the opposite side of the hermaphrodite, only the first turn was noted. Another turn was not counted until after the male completed a turn and continued backing on the opposite side of the hermaphrodite. Vulva location was assayed as follows: the number of times a male passed the vulva before stopping was noted. If the male passed the vulva more than one time before stopping, the male was considered mutant for that behavior.

#### Drug assays

Aldicarb was purchased from Chem. Service (Westchester, PA, USA). For assays, aldicarb was diluted from a 5 mM, or for the assays comparing males and hermaphrodites, a 25 mM, frozen stock solution. Levamisole was purchased from Sigma-Aldrich. Levamisole was diluted from a 50 mM frozen stock solution. All drugs were diluted in ddH20. L4 males were isolated the day before assays. An exception to this is M cell, dorsal body wall, and sham-ablated males, which were first assayed for mating behavior and then after a recovery period were assayed for tail curling in response to drugs. To assay males for tail curling in response to drugs, 500 μl of drug or ddH20 control solution were put in Becton Dickinson 24-well tissue culture plates. Three to six males were assayed at one time and the solution used was discarded after each assay. Males were assayed immediately, and then at 2.5-minute intervals for the first 10 minutes and then at 5-minute intervals. At each time point, males were examined for 20 seconds. Males were considered positive for each behavior if they curled their tail for >5 seconds. If both types of tail curling were seen during that period only the first response seen was recorded.

### Laser ablation

Laser ablation was done using a VSL-337 nitrogen laser and a Zeiss axioskop using standard protocols [32]. Sham-ablated males were mounted on slides containing sodium azide and recovered at the same time as those that underwent laser ablation. The M cell was ablated in L1 stage animals and successful killing was determined by the presence of crumpled spicules. The dorsal body wall muscles were killed in L3 males.

### Molecular biology

The *unc-29*::dsRed construct was made by amplifying a fragment containing 1529 base pairs upstream of the start of *unc-29 *and having PstI sites designed at the ends of both primers. This was then first sub-cloned into the T-easy vector and then digested with PstI. The PstI fragment was then sub-cloned into the vector PSX77, containing dsRed 2 followed by the unc-54 3' UTR (courtesy of S. Xu), and injected into *pha-1*; *him-5 *males along with a *pha-1 *rescuing plasmid. Three stable lines were examined for expression of *unc-29*::dsRed and all had similar expression patterns.

### Statistics

All statistical tests were done using the GraphPad InStat software. Appropriate tests were chosen based on the recommendations of the program.

## Abbreviations

GABA: gamma-aminobutyric acid; GABAR: gamma-aminobutyric acid receptor.

## Authors' contributions

AJW and PWS conceived the study and designed the experiments. AJW carried out the experiments. AJW wrote the manuscript and PWS provided input and revised the manuscript.
